# Supplementary material for: Content-rich biological network constructed by mining PubMed abstracts
Source: BMC Bioinformatics. 2004 Oct 8;5:147. doi: 10.1186/1471-2105-5-147 (PMC528731; doi:10.1186/1471-2105-5-147)
Supplement: Additional File 2 — The original results of the above study (non-essential files are deleted to keep the file size under the limit set by BMC bioinformatics). [file 1471-2105-5-147-S2.bz2 › chilibotAdditionalFile2/dip20/464ID11259614E10491/html/CXCR3_CXCL9.html]

 


 **CXCR3** and **CXCL9** 
  
Found 192 abstracts in PubMed, retrieved 20.  
 

 What does Google say? 
 PDF only 
| .edu only 

---

**Interactive relationship** (e.g. stimulation, inhibition, etc)

**Neutral relationship**- Human microvascular endothelial cell line 1 HMEC 1, transfected with either the known  **CXCR3**  renamed  **CXCR3**  A or  **CXCR3**  B, bound  **CXCL9** , CXCL10, and CXCL11.  Ref: 12782716 J Exp Med, 2003
- AIM To assess the immunological role of human milk by analysing the concentrations of interferon gamma inducible protein of 10 kda IP 10  [ **CXCR3** ]  and monokine induced by interferon gamma MIG  [ **CXCL9** ]  in human milk from mothers of preterm and term infants.  Ref: 12856973 Acta Paediatr, 2003
- Interferon gamma inducible protein 10 IP10  [ **CXCR3** ]  and monokine induced by interferon gamma MIG  [ **CXCL9** ]  positive H& ; RS cells were detected in all 10 HL MC but only in 5 of 10 cases of HL NS and 2 of 9 cases of HL like ATLL.  Ref: 12866030 Int J CancerInt J Cancer, 2003
- Expression of CD4, CD8alpha, CD40, CD86, CD28, CD31 and vascular endothelial growth factor was not different between the two tumor groups but expression of CD40 ligand, CD80, NK1.1 and  **CXCR3**  was relatively lower in A11 Mig  [ **CXCL9** ]  tumors.  Ref: 12792744 Oncol Rep< MedlineTA>Oncol Rep, 2003
- The chemokine receptor  **CXCR3**  is predominantly expressed on T lymphocytes, and its agonists  **CXCL9** , CXCL10 and CXCL11 are IFN gamma inducible chemokines that promote Th1 responses.  Ref: 12884299 Eur J Immunol, 2003
- METHODS Using a semiquantitative polymerase chain reaction method, the authors analyzed the expression of chemokine mRNA coding for interferon IFN gamma induced protein 10 IP 10  [ **CXCR3** ]  and monokine induced by IFN gamma Mig  [ **CXCL9** ]  in a pool of human aortic endothelial cells.  Ref: 12698106 Transplantation, 2003
- DESIGN AND PATIENTS Immunocytochemistry and reverse transcriptase polymerase chain reaction RT PCR amplification were used to analyse the expression of monocyte chemoattractant protein MCP 1, RANTES regulated upon activation, normal T cell expressed and secreted, macrophage inflammatory protein MIP 1alpha, MIP 1beta, interferon IFN gamma inducible protein IP 10  [ **CXCR3** ]  and monokine induced by IFN gamma Mig  [ **CXCL9** ]  in thyroid tissue from patients with Hashimoto s thyroiditis n = 4, Graves disease n = 6 and nonautoimmune multinodular goitre n = 4.  Ref: 12864798 Clin Endocrinol (Oxf), 2003
- An Alternatively Spliced Variant of  **CXCR3**  Mediates the Inhibition of Endothelial Cell Growth Induced by IP 10  [ **CXCR3** ] , Mig  [ **CXCL9** ] , and I TAC, and Acts as Functional Receptor for Platelet Factor 4.  Ref: 12782716 J Exp Med, 2003

**Non-interactive relationship** (e.g. studied together, co-existance, homology, etc.)

- Assessment of cytokine production at the tumor site showed an interdependence of IFNgamma, MIG  [ **CXCL9** ]   **CXCL9**  and IP 10  [ **CXCR3** ]  CXCL10.  Ref: 12740040 Mol Cancer< MedlineTA>Mol Cancer, 2003
- Using an HBV transgenic mouse model, a reduction of hepatic infiltration and liver disease was achieved recently by administration of antibodies directed against the interferon gamma inducible chemokine Mig  [ **CXCL9** ]  and against IP 10  [ **CXCR3** ] .  Ref: 12794718 J Med Virol, 2003
- IP 10  [ **CXCR3** ]  and MIG  [ **CXCL9** ]  mRNA expression levels in cellular components of human milk were determined by RT PCR.  Ref: 12856973 Acta Paediatr, 2003
- Potential molecular differences between early and late rejection grade > =2 episodes were examined by testing chemokine and receptor gene expression.METHODS Expression of inducible protein IP 10  [ **CXCR3** ] , monokine induced by IFN gamma Mig  [ **CXCL9** ] , interferon inducible T cell alpha chemoattractant I TAC, regulated on activation normal T cell expressed and secreted RANTES, and their receptors  **CXCR3**  and CCR5 was tested in 60 endomyocardial biopsies from 24 patients using quantitative Taqman real time polymerase chain reaction PCR.  Ref: 12829909 Transplantation, 2003
- RESULTS We assessed the importance of IFNgamma, IP 10  [ **CXCR3** ]  CXCL10 and MIG  [ **CXCL9** ]   **CXCL9**  in SLC CCL21 therapy.  Ref: 12740040 Mol Cancer< MedlineTA>Mol Cancer, 2003
- The chemokines  **CXCL9**  Mig  [ **CXCL9** ] , CXCL10 IP 10  [ **CXCR3** ] , and CXCL11 I TAC regulate lymphocyte chemotaxis, mediate vascular pericyte proliferation, and act as angiostatic agents, thus inhibiting tumor growth.  Ref: 12782716 J Exp Med, 2003
- In double staining,  **CXCR3**  Mig  [ **CXCL9** ]  coexpressing lymphoma cells were abundant in high MALT of the stomach and thyroid, but rare in other subtypes.  Ref: 12688353 Leuk Lymphoma, 2003
- CCR3 functional responses are regulated by both  **CXCR3**  and its ligands  **CXCL9** , CXCL10 and CXCL11.  Ref: 12884299 Eur J Immunol, 2003
- CONCLUSION We postulate that TFC may play a role in the pathogenesis of autoimmune thyroid disease as they are able to express the chemokines MIP 1alpha, MIP 1beta, MCP 1, RANTES, IP 10  [ **CXCR3** ]  and Mig  [ **CXCL9** ]  that would promote the infiltration of immune cells into the thyroid gland.  Ref: 12864798 Clin Endocrinol (Oxf), 2003
- Interferon gamma inducible chemokines  **CXCL9**  Mig  [ **CXCL9** ] , CXCL10 IP 10  [ **CXCR3** ] , CXCL11 I TAC, strongly associated to Th1 mediated immune responses, belong to the CXC sub family.  Ref: 12739748 J Endocrinol Invest, 2003
- Lesional keratinocytes also overexpressed the ligands of  **CXCR3** , namely, MIG  [ **CXCL9** ]   **CXCL9** , CXCL10, and I TAC CXCL11.  Ref: 12819030 Am J Pathol, 2003
- Remarkably, primary cultures of human microvascular endothelial cells, whose growth is inhibited by  **CXCL9** , CXCL10, CXCL11, and CXCL4, expressed  **CXCR3**  B, but not  **CXCR3**  A.  Ref: 12782716 J Exp Med, 2003
- Studies of cytokine and chemokine gene expression in PBMCs from eight patients revealed induction of IFN gamma, IP 10  [ **CXCR3** ] , and Mig  [ **CXCL9** ] .  Ref: 12684404 Clin Cancer Res, 2003
- Expression of IP 10  [ **CXCR3** ]  and MIG  [ **CXCL9** ]  genes and proteins in the milk cells as well as in mammary gland epithelial tissues was detected by RT PCR and immunohistochemistry.  Ref: 12856973 Acta Paediatr, 2003
- A statistically significant increase of CXCL10 IP 10  [ **CXCR3** ]  and  **CXCL9**  Mig  [ **CXCL9** ]  expression, in thyroid tissue specimens obtained from subjects affected by Hashimoto s thyroiditis and recent onset Graves disease has been reported.  Ref: 12739748 J Endocrinol Invest, 2003
- CONCLUSION These findings indicate that the full potency of SLC CCL21 mediated anti tumor responses require in part the induction of IFNgamma, MIG  [ **CXCL9** ]   **CXCL9**  and IP 10  [ **CXCR3** ]  CXCL10.  Ref: 12740040 Mol Cancer< MedlineTA>Mol Cancer, 2003
- SLC CCL21 mediated anti tumor responses require IFNgamma, MIG  [ **CXCL9** ]   **CXCL9**  and IP 10  [ **CXCR3** ]  CXCL10.  Ref: 12740040 Mol Cancer< MedlineTA>Mol Cancer, 2003
- Our results indicate a tendency for low grade MALT to contain  **CXCR3**  Mig  [ **CXCL9** ]  lymphoma cells, high grade to contain  **CXCR3**  Mig  [ **CXCL9** ]  and DL to contain CRCR3 Mig  [ **CXCL9** ]  lymphoma cells.  Ref: 12688353 Leuk Lymphoma, 2003
- Expression of chemokine receptor  **CXCR3**  and its ligand, mig  [ **CXCL9** ] , in gastric and thyroid marginal zone lymphomas.  Ref: 12688353 Leuk Lymphoma, 2003
- This review discusses chemokine responses in relation to HSV infection of the cornea emphasizing the role of  **CXCR3**  chemokine signaling by the IFN gamma inducible ligands MIG  [ **CXCL9** ] , IP10  [ **CXCR3** ]  and I TAC and includes discussion of their potential role in immunopathology in the nervous system.  Ref: 12815542 Curr Eye Res, 2003
- Our study was designed to investigate the expression of  **CXCR3**  and its ligand Mig  [ **CXCL9** ] , and their relationships in MALT using immunohistochemistry.  Ref: 12688353 Leuk Lymphoma, 2003
- 1 LARC CCL20 whose mRNA level increased rapidly at 3 h post challenge and then gradually decreased, 2 JE CCL2, MARC CCL7, MIP 1gamma CCL9, monocyte chemoattractant protein MCP 5 CCL12, ELC CCL19 and BRAK CXCL14 whose mRNA levels increased with time and reached the maximum at 6 9 h post challenge, 3 LIX CXCL5, Mig  [ **CXCL9** ]   **CXCL9**  and IP 10  [ **CXCR3** ]  CXCL10 whose mRNA levels increased gradually at least up to 12 h post challenge, and 4 SLC CCL21 whose mRNA level decreased gradually with time after challenge.  Ref: 12644322 Immunol Lett, 2003
- There were significant correlations between MIG  [ **CXCL9** ]  and interferon gamma IFN gamma or IP 10  [ **CXCR3** ]  levels p <.  Ref: 12856973 Acta Paediatr, 2003
- Amplified fragments corresponding to KC, IP10  [ **CXCR3** ] , Mig  [ **CXCL9** ]  1, BCL, MIP 2, SLC, RANTES, MCP 1, C10, and Lptn were obtained, and confirmed by Southern blot and sequencing.  Ref: 12667214 Immunology, 2003
- IP 10  [ **CXCR3** ]  and MIG  [ **CXCL9** ]  protein expression in mammary gland tissues was analysed by immunohistochemistry.  Ref: 12856973 Acta Paediatr, 2003
- CONCLUSION IP 10  [ **CXCR3** ]  and MIG  [ **CXCL9** ]  in human milk, probably derived from milk cells and mammary gland epithelial cells, may contribute to the migration and activation of intestinal T lymphocytes to enhance mucosal immunity during the early neonatal period.  Ref: 12856973 Acta Paediatr, 2003
- We have previously shown that SLC CCL21 mediated anti tumor responses are accompanied by significant induction of IFNgamma and the CXC chemokines, monokine induced by IFNgamma MIG  [ **CXCL9** ]   **CXCL9**  and IFNgamma inducible protein 10 IP 10  [ **CXCR3** ]  CXCL10.  Ref: 12740040 Mol Cancer< MedlineTA>Mol Cancer, 2003
- However, 2 of 5 cases of HL like ATLL with EBV infection and 2 of 2 HL NS with EBV had IP10  [ **CXCR3** ]  MIG  [ **CXCL9** ]  positive H& ; RS cells.  Ref: 12866030 Int J CancerInt J Cancer, 2003
- RESULTS Significant amounts of IP 10  [ **CXCR3** ]  and MIG  [ **CXCL9** ]  were detected in human milk.  Ref: 12856973 Acta Paediatr, 2003
- RESULTS Expression of MCP 1, RANTES, MIP 1alpha, MIP 1beta, IP 10  [ **CXCR3** ]  and Mig  [ **CXCL9** ]  was demonstrated in all Hashimoto s and most Graves thyroid specimens but very little expression was detected in the nonautoimmune goitre samples.  Ref: 12864798 Clin Endocrinol (Oxf), 2003
- In contrast to CXCR4, the expression levels of the chemokine receptor,  **CXCR3** , and the migration of cells in response to  **CXCL9**  and CXCL10 increased after IL 2 treatment.  Ref: 12730102 Blood< MedlineTA>Blood, 2003
- METHODS IP 10  [ **CXCR3** ]  and MIG  [ **CXCL9** ]  levels of colostrum, early milk, mature milk and sera were measured by enzyme linked immunosorbent assay ELISA.  Ref: 12856973 Acta Paediatr, 2003
- High levels of RANTES CCL5, MCP 1 CCL2, IP 10  [ **CXCR3** ]  CXCL10, and Mig  [ **CXCL9** ]   **CXCL9**  simultaneously with mononuclear cell infiltration in the lungs was found.  Ref: 12875978 Am J Pathol, 2003
- In vivo depletion of IP 10  [ **CXCR3** ]  CXCL10, MIG  [ **CXCL9** ]   **CXCL9**  or IFNgamma significantly reduced the anti tumor efficacy of SLC CCL21.  Ref: 12740040 Mol Cancer< MedlineTA>Mol Cancer, 2003
- The concentrations of IP 10  [ **CXCR3** ]  and MIG  [ **CXCL9** ]  in colostrum and early milk were significantly higher than those of sera from healthy controls or lactating mothers.  Ref: 12856973 Acta Paediatr, 2003
- RESULTS Mig  [ **CXCL9** ]  and IP 10  [ **CXCR3** ]  were expressed in human endothelial cells both after IFN gamma treatment and after PBMC co culture.  Ref: 12698106 Transplantation, 2003
- The biopsies were taken in the first 3 months or from the 9th to the 12th month following transplantation.RESULTS IP 10  [ **CXCR3** ] , Mig  [ **CXCL9** ] , RANTES,  **CXCR3** , and CCR5 expression levels were increased in the later versus earlier biopsies P< ; =0.01 despite no change in histologic rejection grade status.CONCLUSION These results demonstrate significantly increased expression of T cell chemoattractants in heart allografts during later rejection when compared with episodes occurring shortly after transplantation.  Ref: 12829909 Transplantation, 2003
